# Cognitive-Enhancing Effect of a Hydroethanolic Extract of *Crinum macowanii* against Memory Impairment Induced by Aluminum Chloride in BALB/c Mice

**DOI:** 10.1155/2018/2057219

**Published:** 2018-10-04

**Authors:** Mohammed S. Jilani, Dexter Tagwireyi, Louis L. Gadaga, Charles C. Maponga, Cosmas Mutsimhu

**Affiliations:** ^1^Drug and Toxicology Information Service (DaTIS), School of Pharmacy and Department of Clinical Pharmacology, College of Health Sciences, University of Zimbabwe, P.O. Box A 178, Avondale, Harare, Zimbabwe; ^2^School of Pharmacy, University of Zimbabwe, P.O. Box MP167, Mount Pleasant, Harare, Zimbabwe

## Abstract

*Crinum macowanii* is a bulbous plant indigenous to many parts of Southern Africa. Extracts of *C. macowanii* have gained interest since the discovery of various alkaloids, few of which possess acetylcholinesterase inhibitory activity. The present study was performed to evaluate the effect of a crude hydroethanolic extract of *C. macowanii* against aluminum chloride-induced memory impairment in mice using the Morris water maze and the novel object recognition task. *C. macowanii* (10, 20, and 40 mg/kg p.o) was administered daily for five weeks, while donepezil (3 mg/kg p.o) was used as the positive control. *C. macowanii* at a dosage of 40 mg/kg showed a significantly lower escape latency than the negative control (*P* < 0.0001) and was found to be comparable to donepezil 3 mg/kg in the Morris water maze test. *C. macowanii* at 40 mg/kg exhibited a significantly higher discrimination index than aluminum chloride-treated mice in the novel object recognition task. The results may support the usefulness of *C. macowanii* in the management of dementia and related illnesses.

## 1. Introduction

Alzheimer's disease (AD) is a chronic neurodegenerative disease characterized by cognitive dysfunction, psychiatric, and behavioral disturbances [1]. Hallmark pathological characteristics of AD include the deposition of amyloid-*β* (A*β*) plaques and neurofibrillary tangles in the brain accompanied by synaptic dysfunction [2]. This is the amyloid hypothesis, which postulates that amyloid deposits in the brain are the main cause of AD [3], while the Tau hypothesis suggests that abnormalities in the hyperphosphorylated tau protein are the cause of AD [4]. Early-onset familial Alzheimer's disease is an uncommon form of AD caused by a mutation in one of at least three genes: PSEN1, APP gene, and PSEN2 [5]. Oxidative stress also appears to be a major cause of AD-related pathologies caused by oxidative reactive species [5]. AD is incurable, and presently, there is no treatment that can retard the progression of the disease [6]. The cholinergic hypothesis concludes that the loss of cholinergic function results in memory loss and disruption of cognitive function [7, 8].

Current treatment of AD revolves around the increase in cholinergic transmission between the synaptic cleft by the use of acetylcholinesterase inhibitors (AChEi). These drugs include galantamine, rivastigmine, and donepezil [9]. These medications do not slow the progression of the disease; however, they do provide a modest but consistent improvement in functional and cognitive ability [10]. Since rivastigmine and galantamine are both related to natural products [11], there may still be a benefit in investigating more naturally occurring compounds in order to discover other and perhaps more effective AChEi.

The Amaryllidaceae family contains 1310 species which are widely distributed, and *Crinum* is an important genus of this family [12]. Over the years, alkaloids have been identified as the main active constituents of interest in *Crinum* species. These include lycorine-, crinine-, belladine-, galanthamine-, lycorinine-, and tazettine-type alkaloids [12–14].

*Crinum macowanii* (CM), a member of the *Crinum* genus, is known as *umduze* or *dururu* in the southern African languages of isiNdebele and Shona, respectively, and has a variety of medicinal uses [15]. Various alkaloids which have been isolated from CM include lycorine, 1-O-acetyllycorine, crinine, powelline, crinamine, crinamidine, 3-O-acetylhamayne, 1-epideacetylbowdensine, and cherylline [16]. Some of these alkaloids such as lycorine have been reported to possess acetylcholinesterase (AChE) inhibitory activity [17] and hence could be of benefit in treating dementia. In fact, a recent study reported that a crude hydroethanolic extract of CM had nootropic-like activity in a scopolamine-induced amnesia BALB/c mouse model [18]. However, while the scopolamine AD mouse model used in the aforementioned study [18] is commonly used for assessing nootropic-like activity in rodents, it only assesses cognitive enhancement resulting from the increase in the neurotransmitter—acetylcholine. Yet evidence in the literature suggests that AD is primarily caused by neurodegeneration. Thus, it is possible that the results seen in the study could not be replicated in a model which exhibits neurodegeneration. There is, therefore, a need to validate the results seen by Mugwagwa and colleagues [18] in a model that may be closer to AD. Hence, in this study, we have investigated the possible nootropic effects of CM using the aluminum chloride-induced dementia mouse model. This model not only causes learning memory impairment and cognitive decline in mice but also results in neurodegeneration [19–21].

In order to investigate these effects, the Morris water maze (MWM) and novel object recognition (NOR) test protocols were used. In addition to the behavioral tests, we also investigated the AChE inhibitory and free radical scavenging activity.

## 2. Materials and Methods

### 2.1. Plant Collection

Fresh bulbs of *C. macowanii* were collected in November 2014 at the University of Zimbabwe (UZ) grounds, Harare coordinates 17°47′14.5^″^S 31°03′06.4^″^E. The plant sample was authenticated by a taxonomist from the Botanical Gardens and National Herbarium in Harare. A total number of 24 bulbs were collected for this study.

### 2.2. Preparation of Crude Extract

The crude extract was prepared as described by Mugwagwa et al. [18]. Briefly, fresh bulbs of *C. macowanii* were washed with water and peeled. They were dried under a shade for a period of 3 weeks. To ensure complete drying, the scales were further oven-dried at 55 degrees Celsius. The dried scales were reduced to fine powder using a Thomas Scientific Mill Model 4 (Thomas-Wiley Laboratory, USA) with a sieve size of 1 mm. A total of 1.1 kg of dried powder was obtained. The powder was then macerated in aqueous ethanol (70% *v/v*, up to 8 L) (1 : 10 *w/v*). To ensure the extraction of alkaloids, the maceration was continued for 72 hours with occasional shaking at room temperature. The mixture was then filtered to remove the bulky material using a mutton cloth. The finer material was filtered out by the process of vacuum filtration using Merck number 454 filter paper. This solution was concentrated using a Heidolph instrument, which was the Rotavapor 4000 (Heidolph, Germany). The resultant paste was freeze-dried using Heto Lab Equipment, Heto Freeze Dryer 3 (Heto-Holten A/S, Denmark), for 48 hours to obtain 102 g of the powdered crude extract. This powder was stored in a cool dark place in a desiccator.

### 2.3. Animals

Male BALB/c mice (22-37 g) were purchased from the animal house at the University of Zimbabwe. The mice were housed at the animal holding room in the Clinical Pharmacology Department of the University of Zimbabwe and were allowed to acclimatize for a period of one week. A natural hour light/dark cycle at room temperature was maintained, while a standard pellet diet was made freely accessible. Tap water was provided *ad libitum*. Authorization to use laboratory animals for the research project was granted by the Joint Research and Ethical Committee (JREC approval number: JREC/121/15).

### 2.4. Drugs and Reagents

Donepezil hydrochloride (DONZ) (Actavis Limited, India) and crude extract of *C. macowanii* were used. Aluminum chloride (ALCL) was obtained from the School of Pharmacy, University of Zimbabwe. Normal saline was used as the vehicle for both the CM and the DONZ. Acetylcholinesterase enzyme assay kit was purchased from Sigma Aldrich, South Africa. DPPH (2,2-Diphenyl-1-(2,4,6-trinitrophenyl)hydrazyl) and ascorbic acid were obtained from the Biochemistry Department, University of Zimbabwe. The study was partly funded by the researcher and the School of Pharmacy, University of Zimbabwe.

### 2.5. Grouping and Treatment Schedule

Mice were randomly put into six different groups. Tails of each mouse were marked with a nontoxic marker.

The mice were divided into 6 groups with 6 mice in each group. The experimental design was conducted as described by Mugwagwa et al. [18] and Linardaki et al. [22]. The experimental design was as follows:

Group 1: CM 10 mg/kg/day, p.o. + ALCL 50 mg/kg/day p.o. for 5 weeks

Group 2: CM 20 mg/kg/day, p.o. + ALCL 50 mg/kg/day p.o. for 5 weeks

Group 3: CM 40 mg/kg/day, p.o. + ALCL 50 mg/kg/day p.o. for 5 weeks

Group 4: DONZ 3 mg/kg/day, p.o. + ALCL 50 mg/kg/day p.o. for 5 weeks (positive control)

Group 5: water p.o. (vehicle control); for 5 weeks

Group 6: ALCL 50 mg/kg/day p.o. (negative control) 5 weeks

ALCL was given 30 minutes before DONZ and CM extract to avoid possible interactions between aluminum and other substances.

After 5 weeks, the mice were subjected to the MWM and the NOR test. Following the behavioral tests, the acetylcholinesterase enzyme test and the DPPH free radical scavenging test were performed. In addition, FT-IR and LC-MS fingerprinting analysis of the same extract were also conducted.

### 2.6. Behavioral Tests

#### 2.6.1. Morris Water Maze Test (MWM)

The water maze used was a large metallic circular pool painted black on the inside (110 cm diameter and 40 cm in height). Water (20 ± 1°C) was filled to a depth of 30 cm. The water maze area was divided into four equal quadrants, and a colorless escape circular platform (10 cm in diameter and 25 cm in height) was located in the south-west quadrant. The experiment was monitored using a camera which was positioned directly above the pool. The MWM task was performed as described by Bromley-Brits et al. [23]. Briefly, the experiment was conducted over a period of 6 days. Each mouse was gripped at the base of the tail and gently placed in the water, and time was noted for the mouse to find the platform (escape latency). Mice were given 4 trials per day. On day 1, the platform was made visible by the water being 1 cm below the platform. On days 2–5, the platform was submerged below the water. Day 6 was the probe trial in which the platform was completely removed, and the amount of time spent by the mouse in the target quadrant (south-west) was noted. Each animal was allowed 90 seconds to locate the platform. If an animal failed to locate the platform within this period, it was gently guided to the platform. The animal was left on the platform for 5 s upon locating it. The mouse was then dried with a towel and placed back in the cage.

#### 2.6.2. Novel Object Recognition (NOR) Task

The NOR task was conducted as described by Mugwagwa et al. [18] to assess short-term and long-term memory. The apparatus consisted of an open rectangular box made of plastic with the following dimensions: 43 cm × 31 cm × 16 cm. The experiment was monitored by a camera placed directly above the arena. The test consisted broadly of 2 phases: a sample and test phase. During the sample phase, 2 identical objects (white, square container) were placed equidistant from each other. The mouse was placed in the arena for 5 minutes and then placed back in the cage. The process was repeated for all the animals. The apparatus was cleaned with ethanol between each trial interval so as to remove any olfactory clues which could have been left behind during previous trials. The position of the identical and novel objects was interchanged from left to right in order to prevent bias for a particular location. The same procedure was conducted for the test phase; however, one object was replaced by a novel object (green plastic toy bus). After 5 days, the same procedure was repeated except that the test phase was conducted 24 hrs after the sample phase. The time taken to explore an object was noted.

Object exploration was defined as the mouse licking, sniffing, touching the objects with the paws, or if its head was oriented towards the object within a distance of 2 cm.

The following parameters were measured: time (in seconds) spent exploring familiar object (*T*_f_), time (in seconds) spent exploring the novel object (*T*_n_), and total time (in seconds) spent exploring both objects (*T*_f_ *+ T*_n_). The percentage of discrimination index (DI%) was determined using the following equation:

(1) $$\begin{array}{c} DI\left(\%\right)=\left(\frac{T_{n}}{T_{n}+T_{f}}\right)\times 100, \end{array}$$

where DI = discrimination index, *T*_n_ = time (in seconds) spent exploring the novel object, and *T*_f_ = time (in seconds) spent exploring the familiar object.

### 2.7. Biochemical Tests

#### 2.7.1. Acetylcholinesterase Enzyme Activity Test

Three mice from each study group were sacrificed, and their brains were isolated. The acetylcholinesterase enzyme (AChE) activity test was performed using the AChE enzyme kit from Sigma Aldrich, which measures the AChE activity of the brain homogenate. The assay is based on the Ellman method in which thiocholine, produced by AChE, reacts with 5,5¢-dithiobis (2-nitrobenzoic acid) to form a colorimetric (412 nm) product, which is proportional to the AChE activity present. The isolated brain samples were homogenized with 0.1 M phosphate buffer, pH 7.5, followed by centrifugation at 14,000 rpm. The supernatant was used for the assay. The following equation was used to calculate the ACHE activity:

(2) $$\begin{array}{c} AChE\;activity\left(units/L\right)=\frac{A_{2\min}-A_{10\min}}{A_{calib}-A_{blank}}\times n\times 200, \end{array}$$

where 200 = equivalent activity (units/L) of the calibrator when assayed—read at 2 minutes and 10 minutes, *n* = dilution factor, *A*_2min_ = absorbance of the calibrator at 2 minutes, *A*_10min_ = absorbance of the calibrator at 10 minutes, *A*_calibrator_ = absorbance of the calibrator at 10 minutes, and *A*_blank_ = absorbance of the blank at 10 minutes.

#### 2.7.2. DPPH Radical Scavenging Assay

The free radical scavenging effect of CM was examined using the method employed by Doreddula et al. [24]. Briefly, one mL of 0.2 mM DPPH solution in methanol was mixed with the 1 mL extracts of 3.2, 6.3, 12.5, 25, 50, 100, 200, and 400 *μ*g/mL concentrations, respectively. Absorbance was measured at 517 nm after incubation of the mixture in the dark for 20 min. The free radical scavenging activity of CM was determined by comparing its absorbance with that of a blank solution. Ascorbic acid was used as a reference standard.

The ability to scavenge the DPPH radical was calculated using the following equation:

(3) $$\begin{array}{c} DPPH\;radical\;scavenging\;activity\left(\%\right)=\frac{A_{b}-A_{s}}{A_{b}}\times 100, \end{array}$$

where *A*_b_ = absorbance of the blank and *A*_s_ = absorbance of the sample.

### 2.8. Fingerprinting of the Extract of *Crinum macowanii*

Phytochemical fingerprinting of the extract used in this study was done using LC-MS and FT-IR analysis. In addition, rudimentary qualitative phytochemical screening was conducted to identify possible secondary metabolites in the extract.

#### 2.8.1. LC-MS Analysis

LC-MS analysis was carried out using an Agilent HPLC 1260 System, and an Agilent Q-TOF 6530 mass spectrometer was used as the detector for fingerprinting the extract. 1 mg of free dried sample extract was dissolved in 1 mL of methanol : water (50 : 50) by volume. The resulting mixture was transferred into a 5 mL syringe and filtered through a 0.22 *μ*m acrosdisc syringe filter into an HPLC vial for LC-MS/MS analysis. Gradient elution with composition as follows: solvent A: (water with 0.1% formic acid), solvent B: acetonitrile with 0.1% formic acid, and injection volume: 5 *μ*L. Column temperature was maintained at 40°C and with a total run time of 27 minutes. The LC-Q-TOF-MS data was analyzed using Agilent Technologies Mass Hunter Software Version B.07.03 (509).

#### 2.8.2. FT-IR Analysis

A Fourier-transform infrared (FT-IR) spectrophotometer was used to identify the characteristic functional groups in the extract as a further fingerprint. A portion of the freeze-dried sample was placed on the FT-IR plate with a spatula, and the spectrum was read after background subtraction using the Perkin Elmer Analyst software version 10.5.2.

#### 2.8.3. Phytochemical Screening

Rudimentary phytochemical screening was performed as described by Yadav et al. 2014 [25]. Phytochemical tests were performed for tannins, flavonoids, alkaloids, steroids, glycosides, and saponins.

- Alkaloids: test for alkaloids. The crude extract was mixed with 2 mL of 1% HCl and heated gently. Mayer's and Wagner's reagents were then added to the mixture. The turbidity of the resulting precipitate was taken as evidence for the presence of alkaloids
- Steroids: test for steroid. The crude extract was mixed with 2 mL of chloroform and concentrated sulfuric acid was added sidewise. A red color produced in the lower chloroform layer indicated the presence of steroids
- Glycosides: test for glycosides Liebermann's test. The crude extract was mixed with each of 2 mL of chloroform and 2 mL of acetic acid. The mixture was cooled in ice. Carefully concentrated sulfuric acid was added. A color change from violet to blue to green indicated the presence of a steroidal nucleus, i.e., glycone portion of a glycoside
- Saponins: test for saponins. The crude extract was mixed with 5 mL of distilled water in a test tube, and it was shaken vigorously. The formation of stable foam was taken as an indication for the presence of saponins
- Flavonoids: alkaline reagent test. The crude extract was mixed with 2 mL of 2% solution of NaOH. An intense yellow color was formed which turned colorless on the addition of a few drops of diluted acid which indicated the presence of flavonoids

## 3. Data Analysis

All the results were expressed as mean ± SEM. Data for the MWM test were analyzed by analysis of variance (ANOVA) followed by Tukey's post hoc test in GraphPad Instat package version 7.03. Data analysis for the NOR task was evaluated using one-way analysis of variance (ANOVA) followed by Dunnett's Multiple Comparison tests in GraphPad Prism (version 7.03).

Two-way repeated measures (mixed model) ANOVA followed by Bonferroni posttests were used to compare the two objects in the object recognition task in GraphPad Prism (version 7.03). Data for the biochemical tests were analyzed using two-way analysis of variance (ANOVA) followed by post hoc multiple comparisons using Bonferroni tests. *P* < 0.05 was considered significant.

## 4. Results

### 4.1. Morris Water Maze

The effect of *Crinum macowanii* at three doses (10, 20, and 40 mg/kg) on aluminum chloride-induced memory impairment was investigated. The MWM behavioral test was utilized to assess the escape latencies (EL) and time spent in the target quadrant (TSTQ) expressed in seconds.

**Figure 1:**
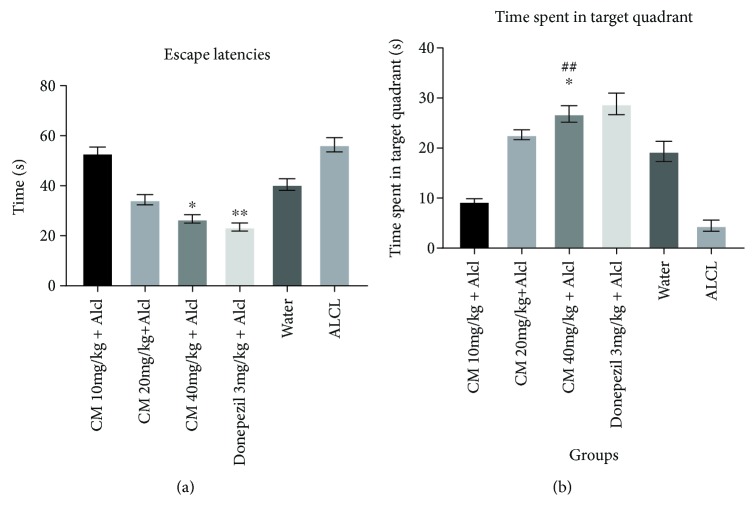
(a) Escape latencies (s) for all groups from day 1 to day 5. Effect of *Crinum macowanii* (10, 20, and 40 mg/kg) on Morris water maze task. ^∗^versus ALCL, *P* < 0.0001, ^∗∗^versus ALCL, *P* < 0.0001. One-way analysis of variance followed by Tukey's post hoc test. (b) Time (s) spent in the target quadrant. ^∗^ versus donepezil, *P* = 0.9538, ^##^ versus ALCL-treated group, *P* < 0.0001.

Results for the EL for all groups are shown in Figure 1(a). There were significant differences in the EL amongst the groups during the course of 5 days (f = 37.43, *P* < 0.0001). There was also a significant difference in the EL between mice in the CM 40 mg/kg group and Aluminum chloride (ALCL) group (*P* < 0.0001) with a mean EL of 26.77 ± 1.67 s and 56.43 ± 2.84 s, respectively. A significant difference was also noted between the DONZ 3 mg/kg group and ALCL group (*P* < 0.0001). No significant difference in EL was seen between CM group at 40 mg/kg and DONZ 3 mg/kg group (*P* < 0.9070). The EL for the ALCL group (mean of 56.43 ± 2.84 s) was significantly higher than the vehicle control group (water, mean of 40.53 ± 2.3 s). The DONZ 3 mg/kg group demonstrated the lowest EL (23.54 ± 1.64 s) followed by the CM at 40 mg/kg group (26.77 ± 1.67 s). Comparing the three dosage groups of CM, the CM at 40 mg/kg group showed the lowest EL followed by the CM at 20 and 10 mg/kg groups.

On day 6, the probe trial was performed wherein the time each mouse spent in the target quadrant (TSTQ) was noted. Results are shown in Figure 1(b). The ALCL group spent the least amount of time in the target quadrant (4.5 ± 1.2 s), while the highest was seen with the DONZ 3 mg/kg group (28.83 ± 2.13 s). No significant difference was seen in the time spent in the target quadrant between DONZ 3 mg/kg and CM 40 mg/kg (*P* = 0.9358). The CM 40 mg/kg treated group showed significantly more time spent in the target quadrant than the ALCL-treated group (*P* < 0.0001).

### 4.2. Novel Object Recognition Test

The NOR test assessed short-term and long-term memory function. It consisted of a sample and test phase. The discrimination index (DI) was calculated for each trial of the test phase to assess the preference for the novel object.

**Figure 2:**
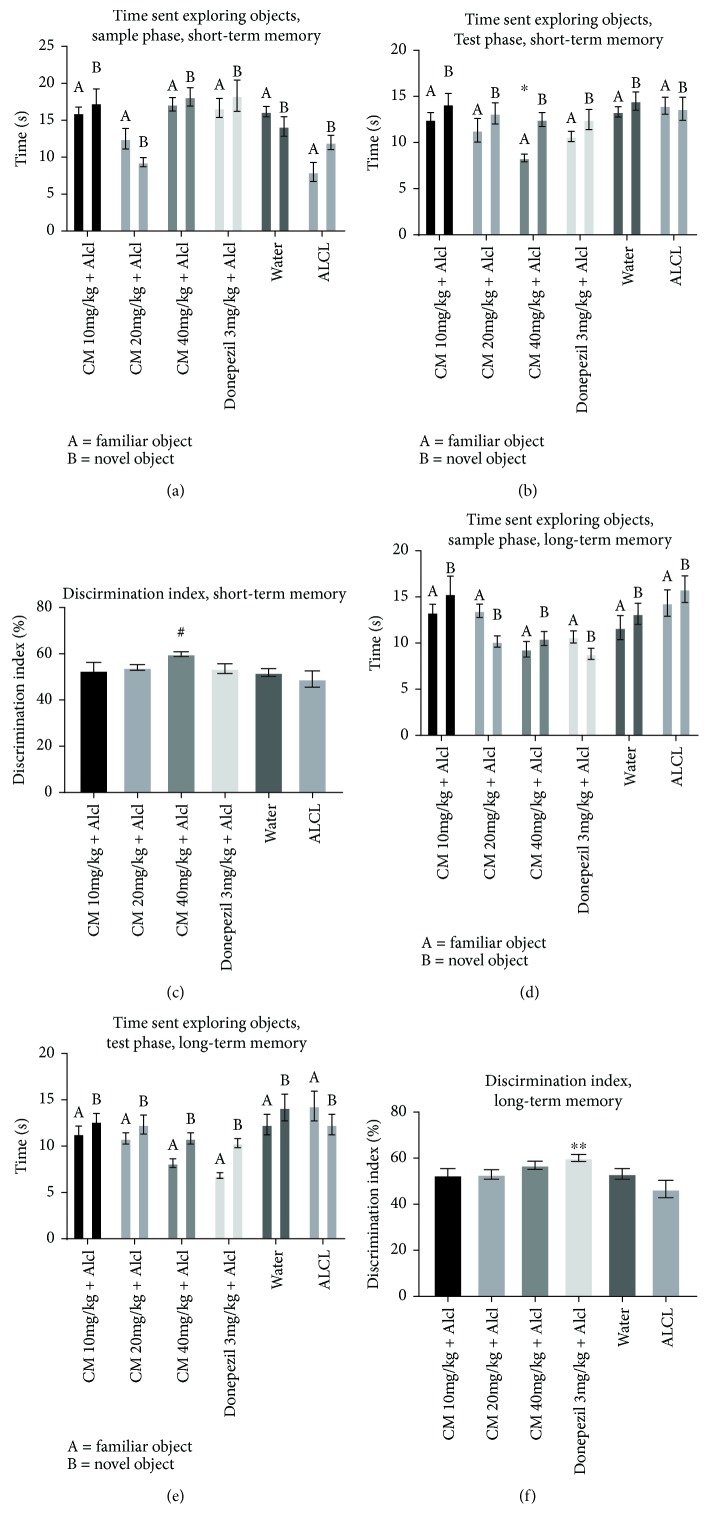
(a) Novel object recognition test, short-term memory, and sample phase. (b) Novel object recognition test, test phase, and short-term memory. (c) Novel object recognition test, discrimination index for short-term memory. Effect of *Crinum macowanii* (CM) (10, 20, and 40 mg/kg) on the short-term memory object recognition task: (a) exploration times in the sample phase, (b) exploration times in the test phase, and (c) discrimination index. ^∗^*P* = 0.0180 novel object versus familiar object, ^#^*P* = 0.0358 versus ALCL. ((a) and (b)) Two-way repeated measures (mixed model) ANOVA followed by Bonferroni posttests. (c) One-way analysis of variance followed by Dunnett's Multiple Comparison tests. (d) Novel object recognition test, sample phase, and long-term memory. (e) Novel object recognition test, test phase, and long-term memory. (f) Novel object recognition test, discrimination index, and long-term memory. Effect of *Crinum macowanii* (CM) (10, 20, and 40 mg/kg) on the long-term memory object recognition task: (d) exploration times in the sample phase, (e) exploration times in the test phase, and (f) discrimination index. (^∗∗^*P* = 0.0071 versus ALCL). ((d) and (e)) Two-way repeated measures (mixed model) ANOVA followed by Bonferroni posttests. (f) One-way analysis of variance followed by Dunnett's Multiple Comparison tests.

The DI for the short-term phase is shown in Figure 2(c). During the short-term phase, there was no significant difference in the DI amongst the groups except between the CM 40 mg/kg and ALCL groups (*P* < 0.0316). The DI for the CM 40 mg/kg group (%DI = 58.97 ± 1.05) was higher than the DONZ 3 mg/kg group (% DI = 53.62 ± 2.1) during the short-term memory task, although this was not significant (*P* = 0.4509). During the short-term memory test, the DI was above 50% for all groups except for the ALCL-treated group (% DI = 49.1 ± 3.55). The same was observed for the long-term memory test (% DI = 46.58 ± 3.76).

During the long-term memory test shown in Figure 2(f) the DONZ 3 mg/kg treated group showed a significantly higher DI than the ALCL group (*P* < 0.0071).

During the sample phase of the short-term memory test, there was no significant difference in the time spent exploring each identical object (*P* > 0.05). In the test phase of the short-term memory test, CM 40 mg/kg treated mice spent significantly more time exploring the novel object than the familiar object (*P* = 0.0180).

During the sample phase of the long-term memory test, there was no significant difference in the time spent exploring each identical object (*P* > 0.05). The CM 10, 20, and 40 mg/kg, DONZ 3 mg/kg, and the control (water) treated mice spent more time exploring the novel object; however, this difference was not significant. In both test phases for short and long-term memory, the ALCL-treated mice showed more time exploring the familiar object than the novel object. This again was not statistically significant.

### 4.3. Acetylcholinesterase Enzyme Activity Test

**Figure 3:**
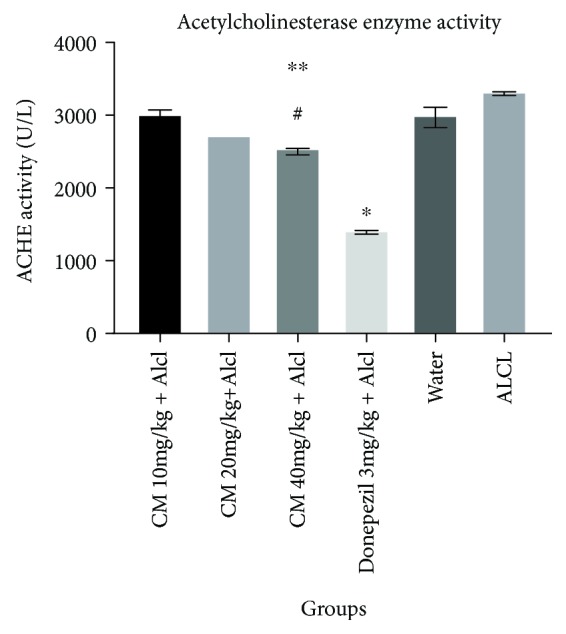
Acetylcholinesterase enzyme activity assay. Effect of *C. macowanii* on AChE activity in the brain homogenate of mice. Values are mean ± SEM. Data was analyzed by two-way ANOVA followed by Bonferroni multiple comparisons test. ^∗^*P* < 0.0001 versus the control group; ^#^*P* = 0.0013 versus the control group; ^∗∗^*P* < 0.001 versus the ALCL-treated group.

The results for the AChE test are shown in Figure 3. The CM-treated group at 40 mg/kg exhibited significantly decreased acetylcholinesterase enzyme (AChE) activity as compared to the ALCL-treated group (*P* < 0.0001) and the control group (*P* = 0.0013). The donepezil-treated group showed the lowest AChE activity (1421 ± 23.75UL) followed by the CM 40 mg/kg treated group (2549 ± 28.65UL). The donepezil-treated group was seen to have a significantly lower AChE activity than the ALCL-treated group (*P* < 0.0001).

### 4.4. DPPH Radical Scavenging Assay

**Figure 4:**
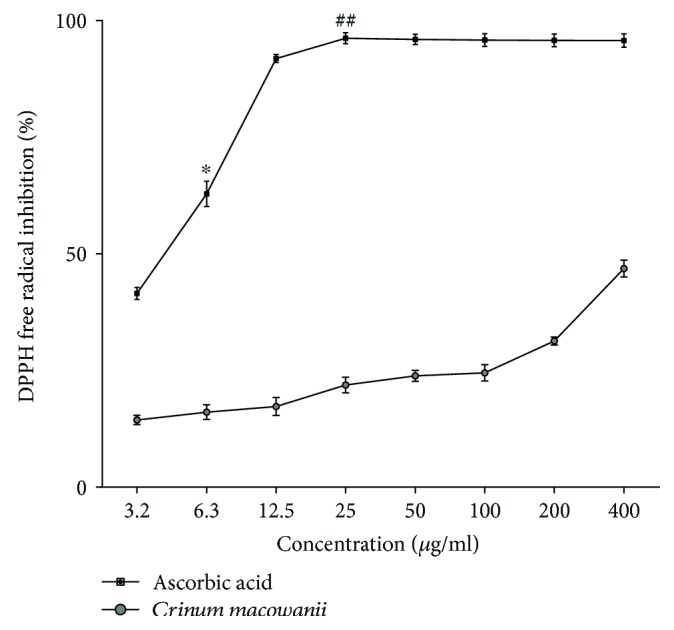
DPPH free radical scavenging activity of a crude extract of *Crinum macowanii*. DPPH radical scavenging activity of *Crinum macowanii* and ascorbic acid. The data are the representative of three independent experiments. Values are mean ± SEM. The data were analyzed by two-way ANOVA followed by Bonferroni multiple comparisons test. CM versus AA, ^∗^*P* < 0.0001; CM versus AA, ^##^*P* < 0.0001.

DPPH radical scavenging activity is shown in Figure 4. A dose-dependent increase in free radical scavenging activity was seen with an increased dose of CM; however, the DPPH free radical scavenging activity of ascorbic acid was significantly higher (*P* < 0.0001) than that of CM at all concentrations.

### 4.5. Results for Fingerprinting

#### 4.5.1. LC-MS Analysis

**Figure 5:**
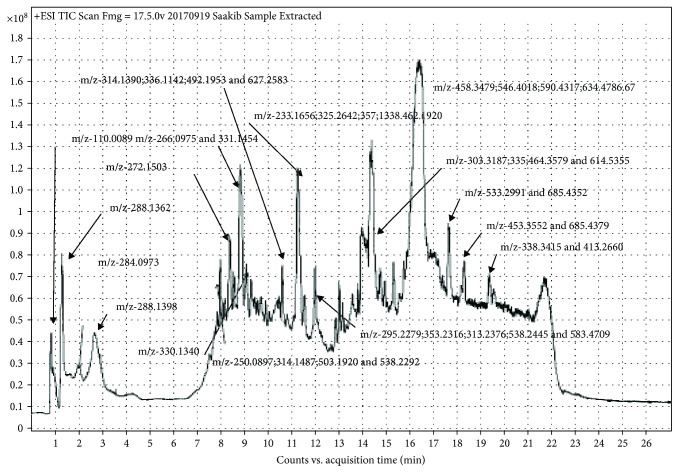
The LC-MS fingerprint of a crude hydroethanolic extract of *C. macowanii*. The LC-MS fingerprint of a crude hydroethanolic extract of *C. macowanii* with prominent peaks.

The results for LC-MS chemical fingerprint are presented in Figure 5. The extract used in this study showed a number of prominent links. As this was meant to only be a fingerprint, no attempt was made at trying to identify the compounds associated with the respective retention times and mass to charge ratios.

#### 4.5.2. FT-IR Analysis

**Figure 6:**
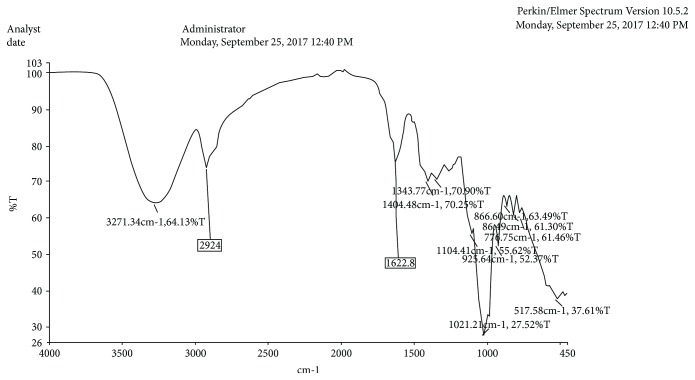
FT-IR spectrum of a hydroethanolic extract of *Crinum macowanii* showing the most prominent peaks.

The FT-IR fingerprint of the compound suggested the presence of various functional groups, which included alkyl, ketone, aldehyde, carboxylic acids, esters, and amide groups. This is presented in Figure 6.

#### 4.5.3. Phytochemical Screening

Rudimentary phytochemical screening of the crude hydroethanolic extract of CM suggested the presence of alkaloids, steroids, glycosides, saponins, and flavonoids.

## 5. Discussion

The current study investigated the effect of a hydroethanolic extract of *Crinum macowanii* bulbs in the aluminum mouse model for Alzheimer's disease using the novel object recognition task and the Morris water maze test for assessing memory function.

Memory function in animal models is generally assessed using various behavioral tasks. We have employed the Morris water maze and novel object recognition task. The MWM test is one of the main behavioral tests used to assess memory function, has the ability to differentiate between spatial and nonspatial memory deficits [26], and can also evaluate learning and relearning [27]. A previous study which evaluated the antiamnesic activity of CM suggested that the extract of CM may be used in the treatment of AD [18]. The study, however, did not utilize the MWM. Moreover, the animal model employed in this current study is known to mimic AD more closely than the scopolamine mouse model.

*β-*amyloid protein *(AβP)* is a peptide resulting from the cleavage of the amyloid precursor protein. This is the basis of the amyloid cascade hypothesis which suggests that the accumulation of *AβP* results in the pathogenesis of AD [28, 29]. It has been demonstrated that the polymerization of *AβP* is enhanced by aluminum [30]. Orally administered aluminum was seen to increase the amount of *AβP* in mice [31]. This suggests that the aluminum mouse model is a relevant animal model for AD. The fact that aluminum has a role to play in the amyloid hypothesis and is an abundant metal which can be commonly ingested by humans makes the aluminum mouse model a relevant model for studying the pathogenesis of AD. In this study, the ALCL-treated mice showed the highest EL and the least time spent in target quadrant, which implied memory impairment due to aluminum.

In the MWM test, a decreased EL during 5 days of trials and an increase in time spent in the target quadrant on the 6^th^ day indicated improved memory function. CM at a dosage of 40 mg/kg showed a significant memory-enhancing effect as compared to the negative control (ALCL) and the vehicle control (water). The CM 40 mg/kg group was also seen to possess similar memory-enhancing effects as the positive control group—DONZ, which is one of the currently approved medications for treating AD. The DONZ 3 mg/kg group showed the lowest EL, although not significantly different than the CM 40 mg/kg group. This suggests that CM at 40 mg/kg was comparable to DONZ 3 mg/kg in improving memory function. The significantly shorter EL of the CM 40 mg/kg treated group as compared to the control group suggests that the *C. macowanii* extract improved memory function in the MWM test against aluminum chloride-induced memory impairment. A dose-dependent decrease in EL was seen in all treatment groups of CM with an increase in dose of the extract.

The probe trial on day 6^th^ was performed by removing the platform from the maze. The time spent in the target quadrant by each mouse was noted and a longer amount of time spent in the target quadrant indicated improved memory function. Similar results were observed during the probe trial, wherein the DONZ group spent the longest time in the target quadrant, but no significant difference was seen in time spent in the target quadrant between the DONZ 3 mg/kg and the CM 40 mg/kg groups. CM 40 mg/kg treated mice spent significantly more time in the target quadrant than the ALCL group. The results of the EL and time spent in the target quadrant indicate that CM extract attenuated aluminum-induced spatial memory impairment.

The novel object recognition test was used to assess short- and long-term memory. The present study evaluated the use of *C. macowanii* extract for its memory enhancing effect against aluminum chloride-induced memory impairment in mice.

Although there was no significant difference in time spent exploring each identical object in the sample phase for short-term memory, significant differences were seen in the test phase.

CM 40 mg/kg treated mice spent significantly more time exploring the novel object than the familiar object (*P* = 0.0180) for the short-term memory task. This was also confirmed by the percentage of discrimination indices which showed a significant difference as compared to the negative control—ALCL. The increase in DI and exploration time of the novel object in mice treated with *C. macowanii* at 40 mg/kg suggested short-term memory enhancing activity. During the long-term memory task for the test phase, no group showed any significant preference for exploring the novel object. Hence, it may be said that none of the doses of *C. macowanii* can be helpful for improving long-term memory against aluminum-induced memory impairment.

Mugwagwa et al. [18] have demonstrated that CM failed to attenuate memory deficits caused by scopolamine in the short-term memory task of NOR. However, CM at high doses showed long-term memory enhancing activity. In contrast, this study has shown that CM was more effective in improving short-term memory rather than long-term memory in the NOR task. The reason for the difference is unclear but may be due to the different mouse models used in each study.

Various studies have reported the presence of alkaloids in CM, while acetylcholinesterase inhibitory activity has also been reported in some [17]. We know that AD is a result of neurodegeneration which results in a decrease in cholinergic transmission in the brain [32]. An effective treatment strategy to improve AChE function is to inhibit the enzyme-acetylcholinesterase which is responsible for the breakdown of acetylcholine, thereby increasing the amount of acetylcholine. CM is an ideal candidate since we have established that the extract does inhibit AChE. The AChE activity test was performed to assess the enzyme inhibitory activity of CM. A higher enzyme activity indicates an inverse relation with the amount of acetylcholine present in the brain. The AChE assay exhibited a dose-dependent decrease in AChE activity in the CM-treated groups. Isolated samples obtained from the brains of the CM-treated groups showed a significantly lower AChE activity than the ALCL-treated group and the control group. The outcome of this study shows that CM has an inhibitory effect on the enzyme—AChE. The AChE activity is likely due to the presence of alkaloids such as lycorine [17].

The DPPH free radical scavenging assay was performed to assess the antioxidant activity of the crude extract of CM. Although a dose-dependent increase of free radical scavenging activity was seen with an increased dose of CM, the activity was significantly less than that of ascorbic acid.

Phytochemical screening chemical tests for the hydroethanolic extract of CM showed the presence of glycosides, alkaloids, saponins, steroids, and flavonoids. The presence of flavonoids may play a neuroprotective role in AD.

The pathogenesis of AD is a complex process resulting from many factors such as ingestion of toxic metals, e.g., aluminum, the decrease in cholinergic transmission, the presence of amyloid protein, and/or neurodegeneration caused by free radicals. These AD-like pathologies may potentially be treated by CM since this study has shown CM to possess AChE inhibitory activity and antioxidant activity. Furthermore, CM has shown to attenuate ALCL-induced memory deficits in the MWM and NOR tasks.

## 6. Conclusion

The hydroethanolic crude extract of *C. macowanii* at a dose of 40 mg/kg showed short-term memory enhancing activity in the NOR task. The same was not demonstrated in the long-term memory task. *C. macowanii* at high doses demonstrated a memory-enhancing effect in the Morris water maze test which was seen to be comparable to that of donepezil. Hence, it may be concluded that *C. macowanii* at high doses ameliorates aluminum chloride-induced memory deficits in mice in the Morris water maze and novel object recognition test and may be of potential benefit in treating AD and related disorders.

## Data Availability

The datasets generated and/or analyzed during the current study are available from the corresponding author on reasonable request.

## Abbreviations

AChE: Acetylcholinesterase
AChEi: Acetylcholinesterase inhibitor
A*β*: Amyloid beta
AD: Alzheimer's disease
ALCL: Aluminum chloride
CM: *Crinum macowanii*
DONZ: Donepezil
DI: Discrimination index
EL: Escape latency
FT-IR: Fourier-transform infrared spectroscopy
LC-MS: Liquid chromatography–mass spectrometry
MWM: Morris water maze
NOR: Novel object recognition.
